# Implementing digital pathology: qualitative and financial insights from eight leading European laboratories

**DOI:** 10.1007/s00428-025-04064-y

**Published:** 2025-03-08

**Authors:** Xavier Matias-Guiu, Jordi Temprana-Salvador, Pablo Garcia Lopez, Solene-Florence Kammerer-Jacquet, Nathalie Rioux-Leclercq, David Clark, Christian M. Schürch, Falko Fend, Sven Mattern, David Snead, Nicola Fusco, Elena Guerini-Rocco, Federico Rojo, Marie Brevet, Manuel Salto Tellez, Angelo Dei Tos, Thomas di Maio, Silvia Ramírez-Peinado, Elizabeth Sheppard, Huw Bannister, Anastasios Gkiokas, Mario Arpaia, Ons Ben Dhia, Nazario Martino

**Affiliations:** 1 Hospital Universitari de Bellvitge and Hospital Universitari Arnau de Vilanova IDIBELL, IRBLLEIDA, University of Lleida, CIBERONC, Lleida, Spain; 2 Pathology Department, Vall d’Hebron University Hospital, Barcelona, Spain; 3 Institut Català de La Salut (ICS), Barcelona, Spain; 4 Cytology and Pathological Anatomy Department, Rennes University Hospital, Rennes, France; 5 Nottingham University Hospitals NHS Trust, HMDN, Dept of Histopathology, City Hospital, Hucknall Road, Nottingham, NG5 1PB UK; 6 Department of Pathology and Neuropathology, University Hospital and Comprehensive Cancer Center Tübingen, Tübingen, Germany; 7 Cluster of Excellence Ifit (EXC 2180) “Image-Guided and Functionally Instructed Tumor Therapies”, University of Tübingen, Tübingen, Germany; 8 Institute of Pathology and Neuropathology, Tübingen University Hospital, Tübingen, Germany; 9 UHCW NHS Trust, Coventry, CV2 2DX UK; 10 Department of Computer Science, University of Warwick, Coventry, UK; 11 European Institute of Oncology IRCCS, Milan, Italy; 12 IIS-Fundación Jiménez Díaz-CIBERONC, Madrid, Spain; 13 Technipath, Dommartin, France & Biwako, Lyon, France; 14 Precision Medicine Centre, Queen’S University Belfast, Belfast, UK; 15 Integrated Patholog Unit, Institute for Cancer Research, London, UK; 16 Department of Integrated Diagnostics, University of Padua, Padua, Italy; 17 Regional Diagnostics AstraZeneca, 6340 Basel, Zug/CH Switzerland; 18 Oncology Digital–Precision Healthcare, AstraZeneca, 08028 Barcelona, ES Spain; 19 Oncology Market Access & Pricing, AstraZeneca, Cambridge, UK; 20 Oncology Diagnostics, AstraZeneca, Cambridge, UK; 21 Alira Health, Basel, Switzerland; 22 Alira Health, Barcelona, Spain; 23 Alira Health, Paris, France; 24 Alira Health, Milan, Italy

**Keywords:** Digital pathology, Economic outcomes, Workflow impact, Pathologist perception

## Abstract

Digital Pathology (DP) revolutionizes the diagnostic workflow. Digitized scanned slides enhance operational efficiency by facilitating remote access, slide storage, reporting and automated AI image analysis, and enabling collaboration and research. However, substantial upfront and maintenance costs remain significant barriers to adoption. This study evaluates DP’s financial and qualitative value, exploring whether the long-term financial benefits justify investments and addressing implementation challenges in large public and private European laboratory settings. A targeted literature review, semi-structured interviews, surveys, and a net present value (NPV) model were employed to assess DP’s impact on clinical practice and laboratory financials. Qualitative findings validate the key benefits of DP, including optimized workflow, enhanced logistics, and improved laboratory organization. Pathologists reported a smooth integration, improved training, teaching, and research capabilities, and increased flexibility through remote work. Collaboration within multidisciplinary teams was strengthened, while case examination efficiency and access to archival slides were notably improved. Quantitative results indicate that DP demonstrates strong financial potential, achieving cost recovery within 6 years. DP investment results in a 7-year NPV of + €0.21 million (m) driven by increased productivity and diagnosis volumes. Although the high upfront costs for scanners, training, and system integration pose a significant barrier to the adoption of DP, larger institutions are better positioned to leverage economies of scale. This study underscores the importance of sustained financial support to cope with the initial investment and regional collaboration in driving widespread adoption of DP. Expanding reimbursement policies for pathology procedures could significantly reduce financial barriers.

**Supplementary Information:**

The online version contains supplementary material available at 10.1007/s00428-025-04064-y.

## Introduction

Pathology is a cornerstone of precision medicine as it plays a critical role in enabling accurate disease diagnosis and guiding treatment decisions through the integration of histopathology and biomarker testing [1, 2]. The increasing complexity of diagnostic tasks in pathology, driven by the need for additional morpho-biological information to support personalized patient management, and combined with a global shortage of pathologists, poses a significant challenge to realizing the full potential of personalized medicine [3], primary research. In this context, DP provides transformative solutions by optimizing workflows, enabling collaboration, and addressing the demand for highly specialized diagnostics while maintaining acceptable turnaround times (TAT) [4–12].

Digital pathology facilitates remote access and streamlined storage and analysis of slides via specialized software solutions. These advancements support cross-laboratory and international collaboration, remote consultations, and progress in research [4–7, 9–14]. Furthermore, DP lays the foundation for the integration of artificial intelligence (AI)-powered tools, referred to as computational pathology (CP), which enhance diagnostic accuracy, predictive modeling, and treatment planning. These AI applications hold significant promise for advancing pathology and precision medicine [15–19].

However, the implementation of DP in real-world clinical practice faces several challenges, with the financial burden on healthcare institutions being a primary concern [3, 20–22]. Costs may vary depending on multiple factors, such as laboratory size, expertise, operations, selection of technology, and purchasing power, but the initial investment and operational costs remain substantial [3, 21, 23]. All of these costs, combined with unclear short-term benefits, may disincentivize its adoption.

Nonetheless, DP’s long-term financial advantages, including improved operational efficiency (e.g., reduced additional IHC orders, higher case volumes with fewer or stable resources) and workload distribution (e.g., working hour savings, TAT decreases, courier/travel expenses savings), may outweigh these challenges [3, 7, 10, 12, 24].

This research assesses DP’s financial and qualitative value, while addressing key implementation challenges in Europe. It explores whether long-term financial benefits justify investments in DP by analyzing initial costs alongside long-term benefits and considers the perspectives of pathologists and technicians to provide a comprehensive view of DP’s impact on clinical practice.

## Methods

### Targeted Literature Review (TLR)

A TLR was conducted to identify the key aspects of DP implementation, including costs, revenue drivers, and impact metrics. This informed an interview guide validated by international key opinion leaders (KOLs) in Pathology.

### Primary research during and post-laboratory visits

Laboratory visits at eight hospitals across five countries (UK, Germany, France, Spain, Italy) assessed DP impacts. Interviews and surveys collected data on funding, implementation, challenges, and outcomes. Financial data were provided during or post-visits, with anonymized surveys capturing additional qualitative insights (Table 3—Supplementary Material).

### Net Present Value (NPV) model

The model assesses DP’s financial benefits and costs, focusing on NPV over a 7-year forecast for seven pathology departments, with 81.4% (44.5 to 100.0%) of cases digitized. It includes case volumes, reimbursement, personnel metrics, and infrastructure investments across base, best, and worst scenarios. Productivity gains and natural growth (2.06%) [25] are factored in, excluding benefits and investments non-DP-related and asset amortization. A 5% discount factor is applied, and the asset lifespan is in line with the forecast horizon [23, 26, 27], ensuring realistic financial projections based on global trends and primary data (Table 10—Supplementary Material).

## Results

### Qualitative data

Qualitative insights were gathered through interviews with key stakeholders from each laboratory, including pathology department directors, specialist pathologists, pathology residents, technicians, IT staff, and accounting personnel. Additional feedback was obtained via surveys, with responses from 45 pathologists and 47 technicians (Tables 4–9—Supplementary Material).

The laboratories participating in the analysis vary in several aspects, including funding sources (private/public), number of pathologists and technicians, areas of specialization, and timeline of digitization. Despite these differences, all laboratories are part of major academic hospitals, characterized by a high volume of activity and highly skilled workforce. Further details about each laboratory are provided in Table 3 of the Supplementary Material.

### Pre-implementation phase—context, funding, and procurement

Laboratories implemented DP primarily to modernize workflows and to prepare for CP integration. Private sector initiatives were also driven by the need to optimize resources and scale operations. Funding sources included the European Union, charity, and governmental and private sources, often supplemented by hospital budgets.

Procurement methods differed by funding source: public funding involved open tenders, while private funding allowed for direct negotiations with the manufacturers. Most laboratories tested equipment before finalizing selections, focusing on image quality, user-friendliness, interoperability, integration ease, cost, customer service, and peer feedback. Cost reductions were achieved through grouped purchases, extended maintenance periods, and vendor agreements for showcasing DP setups or providing on-site technical support.

### Implementation phase—process and challenges

Laboratories validated DP equipment, and technicians received training from the manufacturers. Most laboratories completed or plan to complete their transition within 6 to 18 months, but adopted various approaches for integrating DP into daily workflows:

- Full and immediate: Three laboratories switched entirely to DP across all specialties at once.
- Gradual, specialty-by-specialty: Four laboratories transitioned gradually, validating each specialty.
- Flexible: One allowed continued microscope use for pathologists preferring a gradual adaptation.

None of the laboratories has digitized cytology, one has digitized in situ hybridization (ISH), and another has digitized immunofluorescence. The transition was generally straightforward for the staff, with reported initial workload increases due to parallel workflows (mostly in laboratories that transitioned gradually), and slower performance during adaptation.

Common challenges included technical integration and change management. Laboratory information system (LIS) integration required high-speed access to whole slide images (WSIs), multi-user functionality, and seamless communication with scanners and storage systems. Integration with staining machines and labeling and tracking systems proved difficult as well, primarily due to the lack of standardized interoperability protocols. Change management challenges arose as some staff resisted adjustments to the new routine practices, although most embraced the transition.

Additionally, storage was an important concern for laboratories. They have adopted different storage options for WSIs based on factors such as slide volume, budget, IT infrastructure, and preferred storage modalities. These options range from on-site and off-site storage to cloud-based systems or centralized storage solutions for laboratory networks, with varying storage capacities to adapt to their needs.

### Impact on laboratory logistics and workflow optimization

Pathologists and technicians reported positive impacts resulting from DP implementation, including reduced risks of slide loss, damage, misfiling, and misreporting. Introducing a labeling and tracking system streamlined workflows by minimizing manual data entry and improving organization. In addition, DP created a more structured workspace, enabled instant access to archived slides for research, teaching, and diagnostics, eliminated the need for slide triaging for each pathologist, as well as the handling and circulation of slide trays, etc.

While DP added tasks such as scanner loading and scanning, laboratories adapted by using high-capacity scanners for overnight bulk scanning and smaller scanners for urgent cases during the day. For multi-site laboratories, DP eliminated delays from physical slide transportation, allowing immediate access to WSIs post-scan. Enhanced quality control (QC) measures ensured smoother diagnostics and higher quality outputs.

Some disadvantages were noted, including reliance on digital systems, where failures could disrupt workflows. Early technical issues, especially with QC and scanning, caused delays, requiring rescanning of slides. Additionally, DP led to implementing extra quality control measures for slides and placed greater emphasis on maintaining high-quality standards throughout the entire process (e.g., block cutting and applying cover slips to glass slides), initially extending processing times. However, as systems became better integrated and personnel more familiar with handling issues, these challenges were mitigated.

### Pathologists’ perceptions of DP: key benefits and challenges

**Fig. 1:**
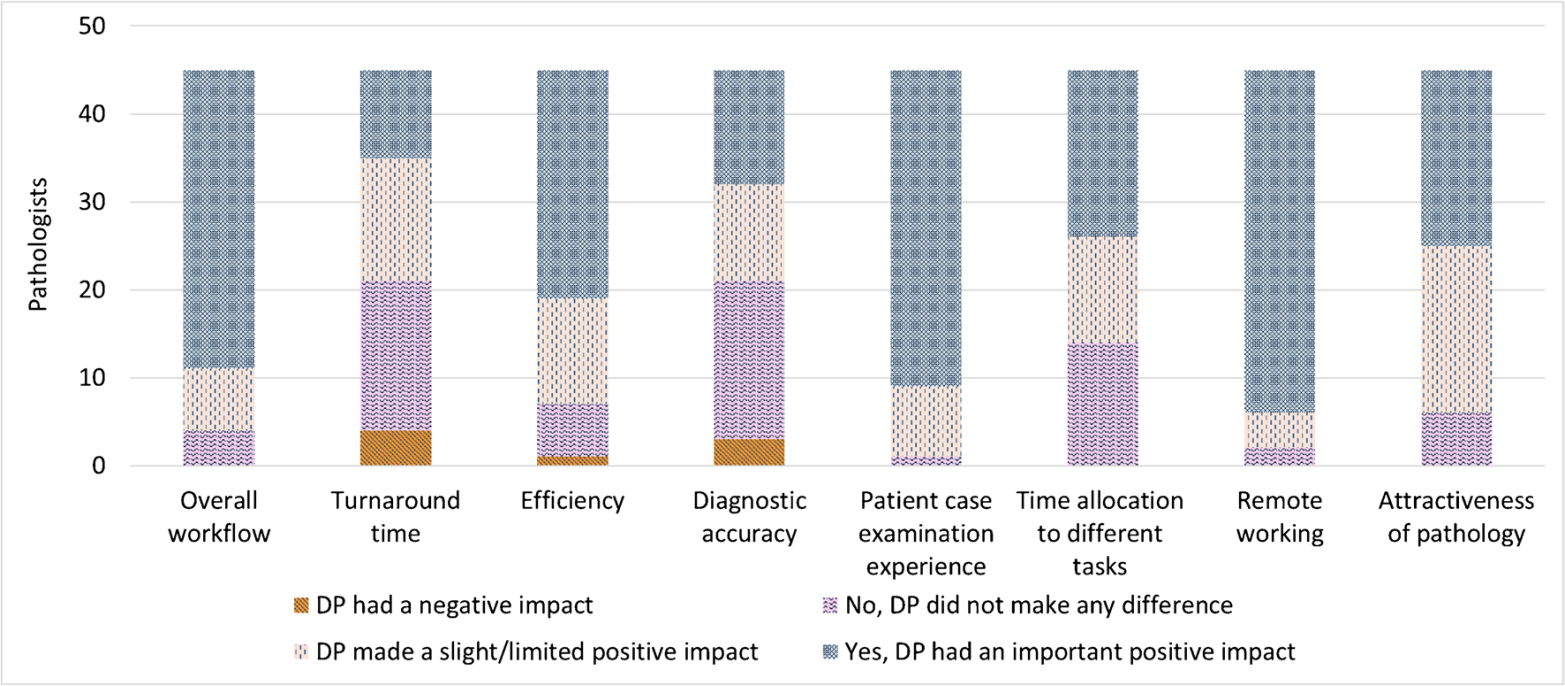
Results of the survey assessing the pathologists’ perceptions of the impact of digital pathology (*n* = 45)

Pathologists reported a highly positive perception of DP (Fig. 1), preferring it over traditional microscopy after a smooth adjustment period of a few weeks to 3 months in most cases.

They noted enhanced daily practice through improved efficiency, patient case examination, and ergonomics (e.g., reduced back strain and visual fatigue). While pathologists were unable to estimate the efficiency gains, faster access to slides, simultaneous viewing of multiple slides, and easier archive access were cited as significant time-saving benefits. Additionally, pathologists highly appreciated the flexibility offered by DP, especially in terms of remote working, as it enhanced their work-life balance.

Digital pathology improved collaboration by enabling second opinions from colleagues during off-hours or across hospitals/countries and simplifying multidisciplinary team (MDT) meeting preparation. However, laboratories without a common digital network faced challenges securely sharing WSIs in formal consultations.

Digital pathology facilitated teaching and research by allowing simultaneous image reviews with trainees and easier access to archival slides. Additionally, DP-enabled laboratories attracted pathologists more easily, addressing workforce shortages with temporary remote support and flexible work options. Remote practicing was particularly valued in the context of service crises such as the recent COVID-19 pandemic [28]. However, remote work flexibility occasionally led to extended working hours.

Despite overall satisfaction, pathologists noted limitations in visualizing specific details, such as depth perception and clarity in certain tissues (e.g., hematology, adipose tissue), although these differences rarely impacted the diagnosis.

### Technicians’ perceptions of DP

**Fig. 2:**
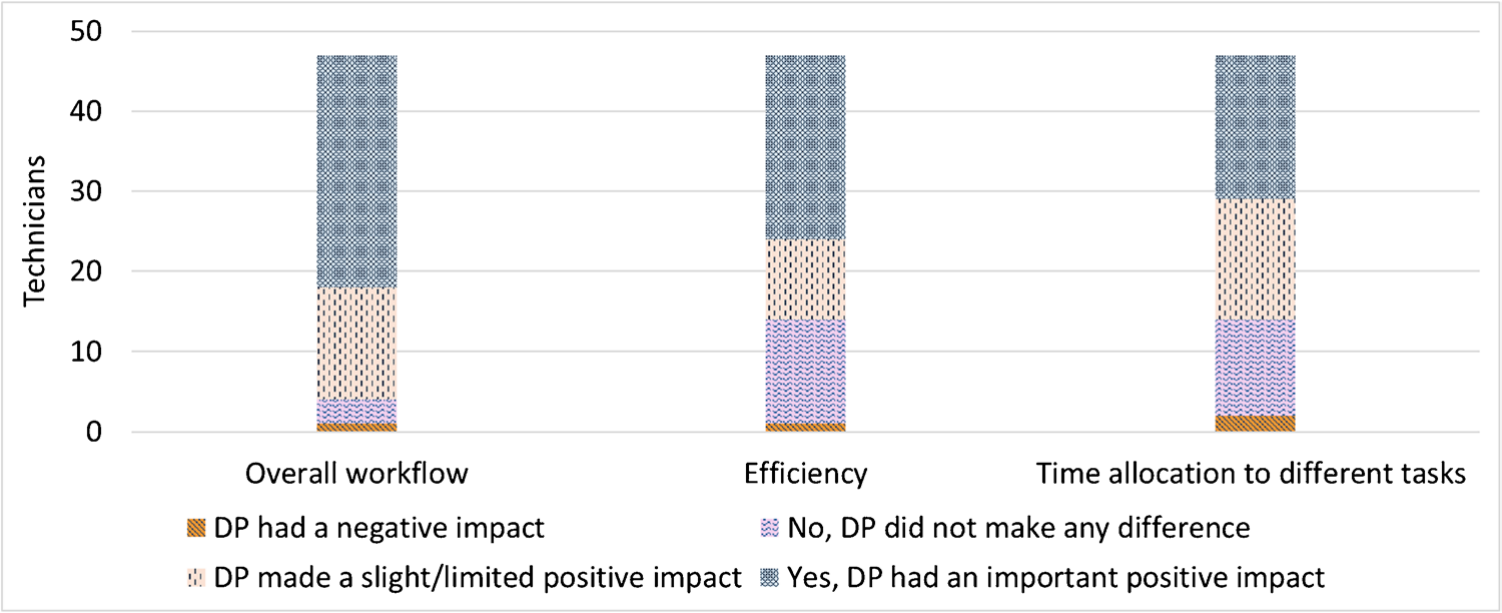
Results of the survey assessing the technicians’ perceptions of the impact of digital pathology (*n* = 47)

Technicians found the DP transition manageable, noting that it replaced old tasks with new ones without significantly increasing workload. Automation of tasks such as data entry and slide triaging streamlined workflows, with reported efficiency gains ranging from 10 to 60% (Fig. 2).

Some technicians felt DP added workload in slide QC and required more careful preparation. Delays due to rescanning were noted as well. Additionally, remote work for pathologists reduced direct interactions with technicians, limiting real-time feedback and engagement.

### Current state of Computational Pathology (CP) implementation

Most laboratories interviewed have limited CP tools, often using only those integrated within the image management systems (IMS) for tasks such as a Ki67 analysis. According to pathologist input, stand-alone CP tools are generally costly. When available, they are funded through research grants or vendor collaborations, making their long-term use uncertain without dedicated reimbursement. Although CP tools could be expensive, many pathologists expressed interest in adopting them but highlighted barriers, such as a lack of reimbursement, high per-use costs, and a greater need for algorithm validation.

### Financial outcomes: long-term financial benefits—NPV model outcomes

In the following section, we present and discuss the results of the model data collection obtained from the participating laboratories. The model synthesizes the input data by averaging inputs across the laboratories. Detailed insights into the data inputs and their distribution are provided in Table 11, located in the supplementary materials section.

### Investments needed

Implementing DP requires substantial financial commitments, including setup costs, recurring expenses, and maintenance. The model captures a 7-year discounted €2.22 million (m) investment in hardware, €1.42 m in software, €1.12 m in IT infrastructure and storage, and €0.32 m in personnel to ensure a sustainable DP workflow during the 7-year period considered (Table 1).

**Table 1:** NPV digital pathology investment needed (discounted figures)

**NPV digital pathology investment needed**	
Data storage and IT infrastructure	€1,118,973
Increase in personnel	€323,807
Software	€1,423,199
Hardware and equipment	€2,221,429
Total investment needed	**€5,087,408**

The total average initial investment, not actualized, amounts to €2.15 m. The cost per case drops from €47.1 at Year 0 or startup to €6.9 at Year 7 or projection end. Efficient scaling and resource use are crucial to sustaining cost reductions and maximizing DP’s economic benefits.

The adoption of DP entails significant upfront costs in technology and infrastructure. Hardware investments, particularly scanners, are the largest expense, comprising a substantial portion of the budget. High-capacity scanners cost approximately €277 thousand (k) each, with five scanners initially required. Additional scanners are added for every 75 k additional slides processed annually (Table 12—Supplementary Materials). Other hardware investments include €4 k per workstation at set up.

The IT infrastructure is crucial for supporting the data-intensive WSI requirements. Over a 7-year period, the yearly cost for data storage and IT systems is, on average, €155 k, with an initial investment of €278 k, covering multi-tier storage solutions and network upgrades. Additionally, annual IT management costs amount to €93 k, highlighting the importance of robust maintenance. Workstations may incur a one-time cost of €4,211, depending on the monitor quality grade and specialized equipment, such as a pathologist’s digital navigation controller for case analysis.

The integration of pathology-viewer software, case managers, and LIS is essential for streamlining workflows and it may cost between €73 k and €137 k annually, respectively, with an additional €276 k for the initial setup.

Operational costs play a crucial role in sustaining DP systems, encompassing some key components, such as labor costs and maintenance. Labor costs involve hiring scanning technicians to manage high-throughput workflows, with each additional full-time equivalent (FTE) costing €50 k annually. Maintenance and IT operations are equally significant, with scanner maintenance costing €65 k per year to ensure reliability and prevent disruptions. Although facility adjustments may be needed for new technologies, none of the settings in this study required structural changes.

### Quantified economical benefits

Implementing DP provides substantial economical and operational benefits, transforming diagnostic workflows. This analysis highlights the key advantages, including increased exam volumes, secondary consultations, workforce efficiency improvements, and equipment cost reductions, delivering a 7-year discounted €5.29 m in total benefits over the forecasted period. These include €4.33 m from higher exam volumes due to productivity gains, €559 K from secondary consultations, €372 k from workforce efficiency improvements, and €32 k from reduced equipment costs (Table 2).

**Table 2:** NPV digital pathology total benefits (discounted figures)

**NPV digital pathology total benefits**	
Increased exam volumes	€4,329,430
Workforce efficiency increase	€371,963
Reduction in equipment	€31,508
Secondary consultations	€559,434
Total benefits	**€5,292,335**

The largest financial benefit comes from increased exam volumes driven by improved productivity.

Case volumes, steered by the natural growth and captured by means of DP, rise steadily from 56 k in Year 0 to 75 k in Year 7, generating economic benefits that grow from €128 k in Year 1 to €1.63 m in Year 7. Revenues per case grow progressively from €1.4 at Year 0 or startup to €24.7 at Year 7 or end of projection, highlighting the scaling potential of DP. Digital pathology enhances throughput and reduces TAT, enabling higher volumes without significant labor or infrastructure increases, thereby maximizing operational output and returns.

Furthermore, DP supports secure sharing of anonymized slides, boosting secondary consultations. Consultation volumes grew from 2785 cases in Year 0 to 5348 by Year 7, with financial benefits increasing from €30 k in Year 1 to €167 k in Year 7.

The implementation of DP enhances workforce efficiency, enabling organizations to handle growing volumes without adding FTEs. This efficiency translates into annual labor cost savings, or redistribution, of 0.20 pathologist FTEs and 0.80 technician FTEs, totaling €372 k over 7 years. The reduction in technician and pathologist FTEs can translate into annual savings ranging from €0 to €57 k, reaching up to €107 k. It is important to note that the reduction in FTEs should not be interpreted as a pure workforce reduction but rather as an opportunity to reallocate time to other laboratory tasks as captured in the qualitative part of the survey. The economic benefit was calculated by multiplying the primary data on FTE reduction with the average annual salaries of pathologists and technicians per FTE.

In addition, DP decreases reliance on optical microscopy, saving costs on microscope replacement and maintenance. The model projects a €32 k in yearly savings over 7 years.

Further considerations, addressing best- and worst-case scenarios, are discussed in the Supplementary Materials.

### Business case

**Fig. 3:**
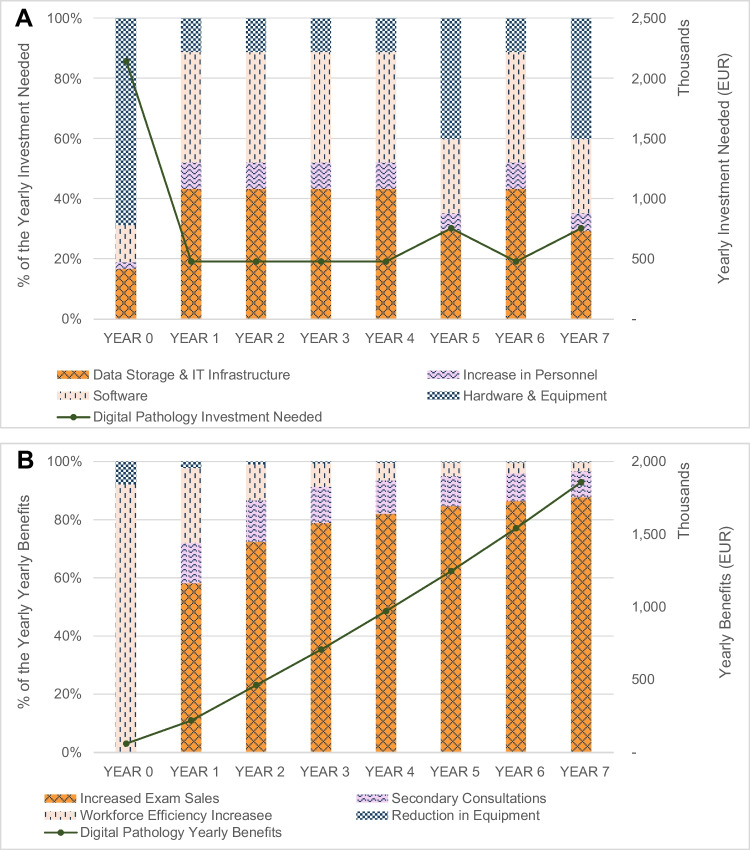
Digital pathology yearly cash flow (thousands EUR) and the proportion of components expressed as a percentage of the yearly investment needed and the yearly benefits. **A** Digital pathology yearly investment needed. **B** Digital pathology yearly benefits

The DP business case shows a steady improvement in cash flow over time, turning positive by Year 3, with the non-discounted cash flow reaching €1.10 m and the discounted cash flow at €0.78 m by Year 7, demonstrating the financial feasibility of DP adoption (Figs. 4 and 5—Supplementary Material). Over a 7-year timeframe, it demonstrates a slightly positive 7-year NPV value of €0.21 m, with actualized economical quantified benefits totaling €5.29 m and an actualized economical investment needed of €5.09 m in the studied setting. Figure 3A, B shows that investment in hardware and equipment accounts for more than 60% in the first year, while the increase in exam sales will drive benefits from Year 1 onwards. The sensitivity analysis (Fig. 6—Supplementary Material) reveals growth and case processing efficiency as the key NPV drivers.

## Discussion

### Interpretation of qualitative and quantitative results

#### Qualitative finding interpretation

The success of transitioning to DP relies not only on the technology itself but on optimizing laboratory processes and managing the transition effectively.

A timely transition helps laboratories fully realize DP’s benefits more quickly, but depending on different laboratory logistics, a staggered or comprehensive rollout approach can be used. Many factors affect the transition duration, including procurement, delivery, implementation, validation, and adoption into the routine workflow. However, hybrid workflows, involving both DP and microscopy, may be less effective as they slow adaptation and increase workload [29]. If a staggered approach is chosen, minimizing the overlap period is essential. This transition phase typically involved substantial process changes, including the standardization and modernization of laboratory operations, enhanced focus on slide preparation, and the need for personnel to become familiar with DP tools, which initially demanded extra time and attention. However, following the integration phase, these adjustments were seamlessly incorporated into the routine practices of the laboratory teams.

Optimizing laboratory processes is key to maximizing DP’s potential. Workflow optimization tools, such as voice recording, speech recognition, and labeling and tracking systems, streamline processes and minimize manual tasks, even though these tools operate independently of DP [29, 30]. Scheduling scanning times, performing QC, and investing in modern equipment further enhance efficiency [29, 31, 32]. A stable Internet connection, user-friendly systems, and high-quality scanners greatly influence laboratory productivity and perceptions of DP [29, 31, 32]. Collaboration for secondary opinions is a key advantage of DP, particularly in networks, such as DigiPatICS or Quirón Salud [33], as these networks allow real-time case sharing and review. However, achieving this level of integration requires careful planning during implementation, including secure network and LIS harmonization. Without this groundwork, sharing slides and patient data between hospitals can be challenging [33].

There are notable disparities among the countries in scope regarding DP implementation, emphasizing the need for tailored strategies, aligned with the unique characteristics of each healthcare system. Pathologists’ willingness to adopt DP is crucial; although some of them can be initially reluctant to adopt DP, most pathologists express high satisfaction after experiencing its benefits [3, 29, 34].

### Quantitative finding interpretation

Digital pathology implementation presents a transformative opportunity to modernize diagnostic workflows and to address inefficiencies [35]. Developing a strong business case is essential for securing investment due to DP’s significant upfront costs [36]. However, the long-term financial and operational benefits make it a compelling option for prepared organizations [21].

This economic analysis demonstrates DP’s financial feasibility, with a 7-year NPV of €0.21 m, and a positive cash flow by Year 3. These findings align with previous studies [7, 37] showing DP’s ability to deliver financial benefits through operational efficiencies and reduced ancillary costs [3, 38].

Key drivers of financial sustainability include increased productivity, higher case volumes, and expanded digital case processing. According to literature, this model demonstrates that DP enables institutions to process growing case volumes without additional FTEs, thereby optimizing time and resources while delivering significant economic benefits [3, 35]. By redistributing workloads and reducing manual processes, DP maintains diagnostic quality while improving efficiency. These labor-related benefits address workforce shortages amid rising global demand for skilled professionals.

Despite these advantages, DP’s substantial upfront costs for hardware, software, storage, and LIS integration remain significant barriers [21]. While large laboratory departments of academic, tertiary hospitals, and public healthcare settings often leverage public funding to offset costs, smaller institutions may struggle, underscoring the need for tailored funding strategies.

Storage represents one of the most significant cost drivers, with expenses expected to rise as the required storage capacity continues to grow. While storing WSIs provides the benefit of rapid access for re-evaluation, retention practices differ across laboratories. Some laboratories do not currently plan on deleting WSIs, while others adopt deletion policies to manage costs. These policies may involve removing WSIs after a set period (e.g., 2 or 6 months or longer) or retaining only those deemed valuable for teaching or research purposes. If needed, as laboratories are required to retain physical slides, re-scanning remains a viable option. However, pathologists have noted a trend toward decreasing storage prices. This reduction could positively impact the financial sustainability of DP, potentially improving its NPV.

Still, DP significantly enhances operational efficiency, enabling pathologists to process more cases with the same resources and achieving an average productivity increase of 7.4% [7, 10, 36, 37]. These improvements stem from streamlined workflows, faster access to digital slides, and eliminating delays from physical slide handling [39]. In addition, this study identifies a 15.30% reduction in turnaround time, emphasizing DP’s efficiency gains [3]. Moreover, DP supports rapid sharing of anonymized cases for secondary consultations, increasing collaborative diagnostics by 19%.

Large tenders, often including multi-year service packages, training, and bundled software, covering case managers, slide viewers, and LIS interfaces, usually involve multiple hospitals or laboratories and can enhance institutions’ bargaining power, significantly reducing costs [33]. Additional savings may come from hospitals covering IT setup and data storage, as these services are typically shared across departments.

### The future of histopathology: computational pathology

There are more than 50 CE-IVD diagnostic CP tools available to diagnostic pathologists [40]. Digital pathology establishes a strong foundation for CP, and pathologists working in a DP ecosystem are generally receptive to computational tools and algorithms that save time and boost efficiency. During laboratory visits, many pathologists emphasized the importance of CP in realizing the full potential of DP, stating, “the main goal of DP is to enable CP.” Supporting research underscores this, as CP algorithms have demonstrated expert-level performance in tasks prone to inter-observer variability (e.g., diagnosis, grading, mitoses enumeration, and subtyping) across therapy areas, such as the breast [8, 41, 42], prostate [43], colorectal [44], ovarian [45], and lung cancer [46–48].

However, CP adoption remains limited. High costs, lack of reimbursement, and the limited trust pathologists have in the current reliability of the algorithm are the main barriers that discourage adoption. Most laboratories access CP solutions through research funding initiatives or strategic collaborations with developers, but broader adoption will require clearer financial incentives and coverage frameworks. To date, most CP tools have been designed to support the pathologist’s decision. As algorithms progressively integrate into routine care and become more diagnostic than supportive [49], prioritization of CP may change.

#### Call to action: recommendations

### Organization into pathology networks

Digital pathology enables telepathology and cross-laboratory collaboration. Networks, such as DigiPatICS, Quiron Salud, and Pathlake, have achieved enhanced scalability, access to subspecialties, data pooling for research and AI training, and cost efficiency through centralized storage and purchasing power [3, 7]. Moreover, establishing laboratory networks could facilitate the adoption of DP in smaller laboratories, by reducing the upfront investment costs, and enabling them to leverage the expertise of larger academic centers. This approach could enhance the quality of care delivery while potentially offering long-term economic advantages. While careful planning is essential, organizing into networks is highly beneficial.

### External funding

Most laboratories implemented DP with external financial support from governmental (e.g., KHZG, Innovate UK), European (e.g., NGEU), or charity-based funding schemes, acting as catalysts for adoption. While our NPV model shows DP is self-sustaining with a positive NPV, smaller laboratories with fewer cases, no network affiliation, and limited funding may face challenges. High initial costs remain a barrier, making continuous financial support from policymakers crucial for wider adoption.

### Reimbursement and coverage for CP

Reimbursement of H&E and IHC diagnostics do not account for advanced technologies. Artificial intelligence-driven interpretation of immunohistochemistry could enhance accuracy and biomarker detection but requires additional financial support due to high costs [50]. Expanding reimbursement for such solutions could reduce the financial burden on laboratories and promote access to innovative diagnostics.

### Limitations

The study faced limitations that could affect the finding’s accuracy.

First, it focused primarily on public academic laboratories with high case volumes, access to external funding, and less emphasis on long-term financial benefits when adopting innovation. While two private laboratories were included, both were part of larger networks. Smaller or independent private laboratories may have different outcomes.

Varying levels of DP implementation across laboratories impacted the assessment, as some were still transitioning to digital workflows, potentially skewing the NPV model’s estimated impact. Additionally, limited access to specific data due to confidentiality or unavailable LIS data points occasionally led to reliance on staff insights and publicly available data, introducing estimation-based variability. Due to insufficient data, the model did not include one of the laboratories, resulting in a final total of seven laboratories for the quantitative results and eight for the qualitative results.

Economic benefits from increased case volumes via DP were calculated using average reimbursement tariffs from primary interviews. While these increases may not directly boost budgets or revenue, they underline DP’s ability to handle higher workloads with constant or reduced resources, addressing challenges, such as pathologist and technician shortages. Informal secondary consultations, common in public institutions, could limit the calculated economic benefits.

## Conclusion

This study confirms that DP delivers significant qualitative and financial benefits, including improved workflow efficiency, enhanced teaching and research opportunities, and increased flexibility through remote work, addressing workforce shortages and fostering collaboration.

Financially, DP boosts productivity with higher diagnosis volumes, secondary consultations, and commercial partnerships, resulting in a slightly positive NPV and long-term gains. However, high upfront investments and operating costs remain substantial barriers, particularly for laboratories with limited financial resources.

Hence, sustained external funding and expanded reimbursement policies are essential to unlock DP’s full potential. Policymakers should prioritize investments in DP and advanced diagnostic tools, such as AI-driven solutions and algorithms to foster innovation, alleviate financial challenges, ultimately leading to improving patient outcomes.

## Supplementary Information

Below is the link to the electronic supplementary material.

Supplementary file1 (DOCX 155 KB)

## Data Availability

The aggregated dataset from all the investigated settings, generated during and/or analyzed during the current study, are available from the corresponding author on reasonable request. Data related to individual settings cannot be shared due to a confidentiality agreement between the laboratories and Alira Health.

## References

[1] Horii R, Akiyama F (2016) Histological assessment of therapeutic response in breast cancer. Breast Cancer 23(4):540–545 24173652 10.1007/s12282-013-0499-6

[2] Schacht V, Kern JS (2015) Basics of immunohistochemistry. J Investig Dermatol 135(3):1–4 25666678 10.1038/jid.2014.541

[3] Hanna MG et al (2019) Implementation of digital pathology offers clinical and operational increase in efficiency and cost savings. Arch Pathol Lab Med 143(12):1545–1555 31173528 10.5858/arpa.2018-0514-OA PMC7448534

[4] Eloy C et al (2021) Digital pathology workflow implementation at IPATIMUP. Diagnostics 11(11):2111 34829458 10.3390/diagnostics11112111 PMC8620597

[5] Fraggetta F et al (2021) A survival guide for the rapid transition to a fully digital workflow: the “Caltagirone example.” Diagnostics 11(10):1916 34679614 10.3390/diagnostics11101916 PMC8534326

[6] Hanna MG, Ardon O (2023) Digital pathology systems enabling quality patient care. Genes Chromosom Cancer 62(11):685–697 37458325 10.1002/gcc.23192 PMC11265285

[7] Ho J et al (2014) Can digital pathology result in cost savings? A financial projection for digital pathology implementation at a large integrated health care organization. Journal of Pathology Informatics 5(1):33 25250191 10.4103/2153-3539.139714 PMC4168664

[8] Ivanova M, et al., 2024 Early breast cancer risk assessment: integrating histopathology with artificial intelligence. Cancers (Basel). **16**(11) 10.3390/cancers16111981 PMC11171409 38893102

[9] Montezuma D et al (2022) Digital pathology implementation in private practice: specific challenges and opportunities. Diagnostics 12(2):529 35204617 10.3390/diagnostics12020529 PMC8871027

[10] Retamero JA, Aneiros-Fernandez J, del Moral RG (2019) Complete digital pathology for routine histopathology diagnosis in a multicenter hospital network. Arch Pathol Lab Med 144(2):221–228 31295015 10.5858/arpa.2018-0541-OA

[11] Schüffler PJ et al (2021) Integrated digital pathology at scale: a solution for clinical diagnostics and cancer research at a large academic medical center. J Am Med Inform Assoc 28(9):1874–1884 34260720 10.1093/jamia/ocab085 PMC8344580

[12] Stathonikos N et al (2019) Being fully digital: perspective of a Dutch academic pathology laboratory. Histopathology 75(5):621–635 31301690 10.1111/his.13953 PMC6856836

[13] Hamilton P et al (2019) Digital and computational pathology for biomarker discovery. In: Badve S, Kumar GL (eds) Predictive biomarkers in oncology: applications in precision medicine. Springer International Publishing, Cham, pp 87–105

[14] Pell R et al (2019) The use of digital pathology and image analysis in clinical trials. J Pathol Clin Res 5(2):81–90 30767396 10.1002/cjp2.127 PMC6463857

[15] Cyrta J, and et al., 2024 Multi-site European study of a fully automated AI solution for HER2 scoring in breast cancer

[16] Kather JN et al (2020) Pan-cancer image-based detection of clinically actionable genetic alterations. Nature Cancer 1(8):789–799 33763651 10.1038/s43018-020-0087-6 PMC7610412

[17] Kather JN et al (2019) Predicting survival from colorectal cancer histology slides using deep learning: a retrospective multicenter study. PLoS Med 16(1):e1002730 30677016 10.1371/journal.pmed.1002730 PMC6345440

[18] Kather JN et al (2019) Deep learning can predict microsatellite instability directly from histology in gastrointestinal cancer. Nat Med 25(7):1054–1056 31160815 10.1038/s41591-019-0462-y PMC7423299

[19] Sajjadi E et al (2023) Computational pathology to improve biomarker testing in breast cancer: how close are we? Eur J Cancer Prev 32(5):460–467 37038997 10.1097/CEJ.0000000000000804

[20] Hanna MG, Parwani A, Sirintrapun SJ (2020) Whole slide imaging: technology and applications. Adv Anat Pathol 27(4):251–259 32452840 10.1097/PAP.0000000000000273

[21] Lujan G et al (2021) Dissecting the business case for adoption and implementation of digital pathology: a white paper from the digital pathology association. J Pathol Inform 12:17 34221633 10.4103/jpi.jpi_67_20 PMC8240548

[22] Williams BJ, Bottoms D, Treanor D (2017) Future-proofing pathology: the case for clinical adoption of digital pathology. J Clin Pathol 70(12):1010–1018 28780514 10.1136/jclinpath-2017-204644

[23] Ardon O et al (2023) Digital pathology operations at a tertiary cancer center: infrastructure requirements and operational cost. J Pathol Inform 14:100318 37811334 10.1016/j.jpi.2023.100318 PMC10550754

[24] Evans AJ et al (2021) Establishment of a remote diagnostic histopathology service using whole slide imaging (digital pathology). J Clin Pathol 74(7):421–424 32611763 10.1136/jclinpath-2020-206762

[25] WHO (2024) Global cancer burden growing, amidst mounting need for services. Available from: https://www.who.int/news/item/01-02-2024-global-cancer-burden-growing--amidst-mounting-need-for-services PMC11115397 38438207

[26] Fang Y-T, Rau H (2017) Optimal consumer electronics product take-back time with consideration of consumer value. Sustainability 9(3):385

[27] Hultkrantz L (2021) Discounting in economic evaluation of healthcare interventions: what about the risk term? Eur J Health Econ 22(3):357–363 33616779 10.1007/s10198-020-01257-x PMC7954734

[28] Arends MJ, Salto-Tellez M (2020) Low-contact and high-interconnectivity pathology (LC&HI Path): post-COVID19-pandemic practice of pathology. Histopathology 77(4):518–524 32516836 10.1111/his.14174 PMC7300838

[29] The Leeds Teaching Hospitals (n.d.) The leeds guide to digital pathology, NHS and University of Leed. Available from: https://www.leicabiosystems.com/sites/default/files/media_document-file/2022-01/Brochure%20-%20Leeds%20Guide%20to%20Digital%20Pathology%20%2818778%20RevA%29.pdf

[30] Hanna MG, Pantanowitz L (2016) Bar coding and tracking in pathology. Clin Lab Med 36(1):13–30 26851661 10.1016/j.cll.2015.09.003

[31] Cheng CL, Tan PH (2017) Digital pathology in the diagnostic setting: beyond technology into best practice and service management. J Clin Pathol 70(5):454–457 28062660 10.1136/jclinpath-2016-204272

[32] Fraggetta F et al (2017) Routine digital pathology workflow: the Catania experience. J Pathol Inform 8:51 29416914 10.4103/jpi.jpi_58_17 PMC5760840

[33] Temprana-Salvador J et al (2022) DigiPatICS: digital pathology transformation of the Catalan Health Institute Network of 8 hospitals-planification, implementation, and preliminary results. Diagnostics (Basel) 12(4):852 35453900 10.3390/diagnostics12040852 PMC9025604

[34] Pinto DG, and et al. (2023) Exploring the adoption of digital pathology in clinical settings - insights from a cross-continent study. Available from: 10.1101/2023.04.03.23288066v1.full.pdf

[35] Baidoshvili A et al (2018) Evaluating the benefits of digital pathology implementation: time savings in laboratory logistics. Histopathology 73(5):784–794 29924891 10.1111/his.13691

[36] Williams BJ et al (2019) Future-proofing pathology part 2: building a business case for digital pathology. J Clin Pathol 72(3):198–205 29549217 10.1136/jclinpath-2017-204926

[37] Griffin J, Treanor D (2017) Digital pathology in clinical use: where are we now and what is holding us back? Histopathology 70(1):134–145 27960232 10.1111/his.12993

[38] Guo H et al (2016) Digital pathology and anatomic pathology laboratory information system integration to support digital pathology sign-out. J Pathol Inform 7:23 27217973 10.4103/2153-3539.181767 PMC4872480

[39] Eccher A et al (2023) Cost analysis of archives in the pathology laboratories: from safety to management. J Clin Pathol 76(10):659–663 37532289 10.1136/jcp-2023-209035 PMC10511949

[40] Geaney A et al (2023) Translation of tissue-based artificial intelligence into clinical practice: from discovery to adoption. Oncogene 42(48):3545–3555 37875656 10.1038/s41388-023-02857-6 PMC10673711

[41] Balkenhol MCA et al (2019) Deep learning assisted mitotic counting for breast cancer. Lab Invest 99(11):1596–1606 31222166 10.1038/s41374-019-0275-0

[42] Jiménez G, and Racoceanu D. 2019 Deep learning for semantic segmentation vs. classification in computational pathology: application to mitosis analysis in breast cancer grading. Front Bioeng Biotechnol. **7** 10.3389/fbioe.2019.00145 PMC6597878 31281813

[43] Nguyen TH et al (2017) Automatic Gleason grading of prostate cancer using quantitative phase imaging and machine learning. J Biomed Opt 22(3):036015 10.1117/1.JBO.22.3.036015 28358941

[44] Pai RK et al (2021) Development and initial validation of a deep learning algorithm to quantify histological features in colorectal carcinoma including tumour budding/poorly differentiated clusters. Histopathology 79(3):391–405 33590485 10.1111/his.14353

[45] Orsulic S et al. (2022) Computational pathology in ovarian cancer. Frontiers in Oncology 12 10.3389/fonc.2022.924945 PMC9372445 35965569

[46] Chen CL et al (2021) An annotation-free whole-slide training approach to pathological classification of lung cancer types using deep learning. Nat Commun 12(1):1193 33608558 10.1038/s41467-021-21467-y PMC7896045

[47] Coudray N et al (2018) Classification and mutation prediction from non-small cell lung cancer histopathology images using deep learning. Nat Med 24(10):1559–1567 30224757 10.1038/s41591-018-0177-5 PMC9847512

[48] Kanavati F et al (2020) Weakly-supervised learning for lung carcinoma classification using deep learning. Sci Rep 10(1):9297 32518413 10.1038/s41598-020-66333-x PMC7283481

[49] Shimizu T et al (2023) First-in-human, phase I dose-escalation and dose-expansion study of trophoblast cell-surface antigen 2–directed antibody-drug conjugate datopotamab deruxtecan in non–small-cell lung cancer: TROPION-PanTumor01. J Clin Oncol 41(29):4678–4687 37327461 10.1200/JCO.23.00059 PMC10564307

[50] Gkiokas A, Sheppard E (2024) Market access landscape for digital and computational pathology in EU4, UK, and Switzerland. Poster presented at: European Conference of Pathology

