# Supplementary material for: Implementing digital pathology: qualitative and financial insights from eight leading European laboratories
Source: Virchows Arch. 2025 Mar 8;487(4):815–26. doi: 10.1007/s00428-025-04064-y (PMC12546502; doi:10.1007/s00428-025-04064-y)
Supplement: Supplementary file 1 — Supplementary file1 (DOCX 155 KB) [file 428_2025_4064_MOESM1_ESM.docx]

# Supplementary Material

The supplementary material in this document contains additional details regarding the methodology and the outcomes of this study. This mainly includes:

- Data on the characteristics of the laboratories in scope: Table 3

| Laboratory | Spain-Laboratory 1 | Spain-Laboratory 2 | Spain-Laboratory 3 | UK-Laboratory 1 | UK-Laboratory 2 | France-Laboratory 1 | Italy-Laboratory 1 | Germany- Laboratory 1 |
| --- | --- | --- | --- | --- | --- | --- | --- | --- |
| Status | Public -University Hospital | Public -University Hospital | Private – University Hospital | Public -University Hospital | Public -University Hospital | Public -University Hospital | Private | Public -University Hospital |
| Year of start of Digitization | October 2022 | November2021 | July  2017 | September 2021 | October 2015 | March 2020 | January 2024 | October 2023 |
| Year of finalization of the digitization | November2022 | December 2021 | March 2018 | July  2024 | December 2019 | May 2021 | Ongoing - Planning on finalizing it by December 2025 | Ongoing – Planning on finalizing it by March 2025 |
| Transition approach* | Full and Immediate | Full and Immediate | Full and Immediate | Gradual, Specialty-by-Specialty | Flexible | Flexible | Gradual, Specialty-by-Specialty | Gradual, Specialty-by-Specialty |
| Number of pathologist (including residents) | 22 | 45 | 30 | 55 | 26 | 13 | 30 | 20 |
| Number of Technicians | 52 | 60 | 49 | 50-60 | 52 | 21 | 39 | 60-80 |

- The surveys used: Appendix 1 – Pathologists’ survey; Appendix 2 – Technicians’ survey
- The results of the survey: Appendix 3
- Detailed overview of the NPV model’s methodology and outcomes: Appendix 4.

**Table 3 – Characteristics of the laboratories included in the analysis**

Notes: *Types of transition approaches: **Full and Immediate:** Laboratory switched entirely to DP across all specialties at once; **Gradual, Specialty-by-Specialty:** Laboratory transitioned gradually, validating each specialty; **Flexible:** Laboratory allowed continued microscope-use for pathologists preferring a gradual adaptation.

## Appendix 1 - Pathologists’ survey

**SECTION 1 – Interviewee Background**

1. What is your role? Please provide subspecialty if applicable (e.g., Pathologist specialized in breast cancer, dermatopathology, prostate cancer, etc.)
   - - [Free text response]
2. At which laboratory/ hospital are you currently employed?
   - - [Free text response]
3. How many years of experience do you have?
   - - [Free text response]

**SECTION 2– Perception of DP**

1. Did you perceive an improvement in the pathology workflow after the integration of DP?
2. DP had a negative impact;
3. No, DP did not make any difference;
4. DP made a slight/limited positive impact;
5. Yes, DP had an important positive impact**.**

1. If your answer to the previous question was yes, in which part of the diagnostic process have you perceived the most significant improvement through using digital pathology? (e.g., slide scanning, image analysis, remote access etc.)
   - - [Free text response]

1. Did you perceive an improvement in the average turnaround time (TaT) before and after DP integration?
   1. DP had a negative impact;
   2. No, DP did not make any difference;
   3. DP made a slight/limited positive impact;
   4. Yes, DP had an important positive impact**.**

1. If your answer to the previous question was yes, how much has approximately the TaT improved in terms of percentage? (estimation)
   - - [Free text response]

1. Did you perceive an overall increase in your efficiency after the integration of DP?
   1. DP had a negative impact;
   2. No, DP did not make any difference;
   3. DP made a slight/limited positive impact;
   4. Yes, DP had an important positive impact**.**

1. If your answer to the previous question was yes, could you provide an estimation in percentage? (e.g., 5-10%, 10-15%)
   - - [Free text response]

1. Did DP allow you to allocate your time differently in your current practice/ different tasks? (i.e., do you feel that DP makes you spend more or less time on certain tasks?)
   - 1. DP had a negative impact;
     2. No, DP did not make any difference;
     3. DP made a slight/limited positive impact;
     4. Yes, DP had an important positive impact**.**
2. If your answer to the previous question was yes, what tasks were you enabled to allocate your gained time to? (e.g., more exceptional cases, not working overtime etc.)
   - - [Free text response]

1. Do you perceive the possibility of remote working as a high value benefit of DP?
   - 1. DP had a negative impact;
     2. No, DP did not make any difference;
     3. DP made a slight/limited positive impact;
     4. Yes, DP had an important positive impact**.**

1. Did you perceive a change in the diagnostic accuracy or the ability to detect subtle or rare features with digital pathology?
   - 1. DP had a negative impact;
     2. No, DP did not make any difference;
     3. DP made a slight/limited positive impact;
     4. Yes, DP had an important positive impact**.**

1. If your answer to the previous question was yes, in what way have you achieved increased diagnostic accuracy? (e.g., fewer misdiagnoses, more confidence in the results etc.)
   - - [Free text response]

1. Are you aware if there have been notable improvements in patient care outcomes or treatment decisions attributed to the use of digital pathology? (e.g. enhanced outcomes due to an earlier diagnosis and treatment initiation)

- [Free text response]

1. Have you perceived a positive impact of DP integration on the attractiveness of pathology for medical students?
   - 1. DP had a negative impact;
     2. No, DP did not make any difference;
     3. DP made a slight/limited positive impact;
     4. Yes, DP had an important positive impact**.**

1. Are there any additional aspects where you saw a positive or negative impact of DP?

- [Free text response]

1. Does the use of DP improve your patient case examination experience? (e.g., working remotely, not looking at the microscope, easier transition from one slide to the other, possibility of parallel examination of slides on the monitor, etc.)
2. DP had a negative impact;
3. No, DP did not make any difference;
4. DP made a slight/limited positive impact;
5. Yes, DP had an important positive impact**.**

1. If your answer to the previous question was yes, can you provide additional examples?
   - - - - [Free text response]

1. Do you feel comfortable conducting primary diagnosis using digital pathology, without the availability of physical slides?
   - - - 1. Not comfortable, I often need to use the physical slides in order to make a diagnosis
         2. Somewhat comfortable but would like to keep the possibility of accessing the physical slides
         3. Comfortable, but I might occasionally need access to physical slides
         4. Very comfortable, I can completely forgo the use of physical slides.

1. How would you describe the learning curve and the steps toward DP integration? (e.g. ease of use of DP equipment, trainings, adaptation to the new workflow…)
   - - 1. not difficult
       2. somewhat difficult
       3. Difficult
       4. very difficult

1. How long after DP implementation did you fully experience its impact on the laboratory’s activity?

- [Free text response]

1. What are the most important barriers that pathology departments face currently when it comes to adopting CP/ AI-based pathology solutions?

- [Free text response]

1. Are you familiar with any AI-based CP solutions?
   - - 1. No, I have never used them,
       2. Somewhat, I used them in the past/rarely used them,
       3. Yes, I use them in my routine practice
2. If your laboratory is not using CP tools, how comfortable will you be with adopting a CP solution into your routine clinical practice?
   - - 1. Not comfortable
       2. Somewhat comfortable
       3. Comfortable
3. If your laboratory has already implemented a CP tool, what do you think are the benefits of adopting CP?
   - - - - [Free text response]
4. What are the most important barriers in adopting CP?
   - - - [Free text response]

##

## Appendix 2 - Technicians’ survey

**SECTION 1 – Interviewee Background**

1. What is your role? Please provide subspecialty if applicable

- [Free text response]

1. At which laboratory/ hospital are you currently employed?
   - - [Free text response]

1. How many years of experience do you have?
   - [Free text response]

**SECTION 2– Perception of DP**

1. Did you perceive an improvement in the pathology workflow after the integration of DP?
   - 1. DP had a negative impact;
     2. No, DP did not make any difference;
     3. DP made a slight/limited positive impact;
     4. Yes, DP had an important positive impact.

1. Did you perceive an overall increase in your efficiency after the integration of DP?
   - 1. DP had a negative impact;
     2. No, DP did not make any difference;
     3. DP made a slight/limited positive impact;
     4. Yes, DP had an important positive impact**.**

1. If your answer to the previous question was yes, could you provide an estimation in percentage? (e.g., 5-10%, 10-15%)

- [Free text response]

1. Did DP allow you to allocate your time differently in your current practice/ different tasks? (i.e., do you feel that DP makes you spend more or less time on certain tasks?)
   - 1. DP had a negative impact;
     2. No, DP did not make any difference;
     3. DP made a slight/limited positive impact;
     4. Yes, DP had an important positive impact**.**

1. Has your workload increased after the implementation of DP? If yes, how? (e.g., more slides to be prepared for scanning, more time spent on quality control of slides, scanner scanning waiting time, etc.)

- [Free text response]

1. Are there any additional aspects where you saw a positive or negative impact of DP?

- [Free text response]

1. How would you describe the learning curve and the steps toward DP integration? (e.g. ease of use of DP equipment, trainings, adaptation to the new workflow…)
   - 1. Not difficult
     2. Somewhat difficult
     3. Difficult
     4. Very difficult.

1. How long after DP implementation did you fully experience its impact on the laboratory’s activity?
   - [Free text response]

##

## Appendix 3 – Survey Results

**Table 4 – Number of responders by country (Pathologists)**

|  | **UK** | **France** | **Spain** | **Italy** | **Germany** | **Total** |
| --- | --- | --- | --- | --- | --- | --- |
| **Number of laboratories** | 2 | 1 | 3 | 1 | 1 | 8 |
| **Number of Responses** | 1 | 9 | 30 | 1 | 4 | 45 |

**Table** **5 – Pathologists’ responses to semi-qualitative questions (Likert scale)**

|  |  | **Perceived Level of DP Impact** | | | |
| --- | --- | --- | --- | --- | --- |
| **Question** | | **DP had a negative impact** | **No, DP did not make any difference** | **DP made a slight/limited positive impact** | **Yes, DP had an important positive impact** |
| **Overall workflow** | 4. Did you perceive an improvement in the pathology workflow after the integration of DP? | 0 | 4 | 7 | 34 |
| **Turnaround time (TaT)** | 6. Did you perceive an improvement in the average turnaround time (TaT) before and after DP integration? | 4 | 17 | 14 | 10 |
| **Efficiency** | 8. Did you perceive an overall increase in your efficiency after the integration of DP? | 1 | 6 | 12 | 26 |
| **Time allocation to different tasks** | 10. Did DP allow you to allocate your time differently in your current practice/ different tasks? (i.e., do you feel that DP makes you spend more or less time on certain tasks?) | 0 | 14 | 12 | 19 |
| **Remote working** | 12. Do you perceive the possibility of remote working as a high value benefit of DP? | 0 | 2 | 4 | 39 |
| **Diagnostic accuracy** | 13. Did you perceive a change in the diagnostic accuracy or the ability to detect subtle or rare features with digital pathology? | 3 | 18 | 11 | 13 |
| **Attractiveness of pathology** | 16. Have you perceived a positive impact of DP integration on the attractiveness of pathology for medical students? | 0 | 6 | 19 | 20 |
| **Patient case examination experience** | 18. Does the use of DP improve your patient case examination experience? (e.g., working remotely, not looking at the microscope, easier transition from one slide to the other, possibility of parallel examination of slides on the monitor, etc.) | 0 | 1 | 8 | 36 |
|  |  |  |  |  |  |
|  | **Question** | **Not comfortable, I often need to have access to physical slides to make a diagnosis** | **Somewhat comfortable but would like to keep the possibility of accessing the physical slides** | **Comfortable, but may occasionally need to access physical slides** | **Very comfortable, I can completely forgo the use of physical slides** |
| **Level of comfort with diagnosing only using DP (no glass)** | 20.Do you feel comfortable conducting primary diagnosis using digital pathology, without the availability of physical slides? | 2 | 0 | 18 | 25 |
|  | **Question** | **Not difficult** | **Somewhat difficult** | **Difficult** | **Very difficult** |
| **Level of difficulty of the DP learning curve** | 21. How would you describe the learning curve and the steps toward DP integration? (e.g. ease of use of DP equipment, trainings, adaptation to the new workflow…) | 35 | 5 | 2 | 3 |
|  | **Question** | **No, I have never used them** | **Somewhat, I used them in the past/rarely used them** | **Yes, I use them in my routine practice** |  |
| **Familiarity with AI-based CP solutions** | Are you familiar with any AI-based CP solutions? | 16 | 17 | 10 |  |
|  | **Question** | **Not comfortable** | **Somewhat comfortable** | **Comfortable** |  |
| **Expected level of comfort with the adoption of CP** | 26. If your laboratory is not using CP tools, how comfortable will you be with adopting a CP solution into your routine clinical practice? | 1 | 11 | 21 |  |

**Table 6 – Summary of the pathologists’ responses to qualitative questions (Free text)**

| **Questions and Answers** |
| --- |
| **Improvement in the workflow**  5. If your answer to the previous question was yes, in which part of the diagnostic process have you perceived the most significant improvement through using digital pathology? (e.g., slide scanning, image analysis, remote access etc.) |
| **Image Analysis and Diagnostics:**   - Enables a detailed image analysis for an improved histopathological diagnosis. - Facilitates tumor margin measurement and molecular assessments. - Integration with tools for marker quantification and automated evaluations (e.g., Ki67).   **Remote Access:**   - Allows remote case consultations and remote working. - Facilitates obtaining second opinions from specialized pathologists in other hospitals or regions. - Simplifies remote meetings and staff coordination.     **Efficiency and Speed:**   - Speeds up access to cases, case review (e.g. due to lower magnification than the microscope, faster access to slides with no need to have the physical slides, seeing multiple slides in parallel), and workflow management. - Enhances availability and organization of slides and cases.   **Collaboration and Sharing:**   - Improves case-sharing capabilities across institutions and within networks. - Facilitates communication in multidisciplinary meetings (MDTs).   **Traceability and Safety:**   - Improves case traceability, consistency, and transparency in processes. - Enhances patient safety by standardizing staining and cutting quality (reduced risk of errors and improved diagnostic process).   **Workflow Optimization:**   - Automates processes and reduces manual tasks. - Voice dictation during image analysis and streamlines reporting protocols. - Facilitates access to archival slides - Improved quality of the slides (cutting, and staining) - Improved patient case management (reduces risk of mixing-up slides and patients, easier access to previous slides and reports) - Better organization of the laboratory - Consistency and transparency of the process (pathologists can track at what stage a specific sample is)   **Teaching, Training, and research:**   - Facilitates case reviews with residents. - Facilitates preparation of presentations and submissions for conferences. - Useful for teaching and for preparing conferences. - Easier access to slides for research (i.e. archival slides)   **Ergonomics & Accessibility:**   - Provides better viewing capabilities, including low-magnification visualization and integration of diagnostic tools. - Improves sample accessibility and visualization in all dimensions. |
| **Turnaround Time**  6. Did you perceive an improvement in the average turnaround time (TaT) before and after DP integration? |
| - Difficult to evaluate. - May depend on the type of samples - Increased TAT by 1 day because of scanning - Less than 1 day - Different estimations of the percentage of TAT reduction (generally between 5-20% TAT reduction) |
| **Efficiency**   1. If your answer to the previous question was yes, could you provide an estimation in percentage? (e.g., 5-10%, 10-15%)*   **The responses were not used to inform the efficiency increase in the NPV model.* |
| - **Number of responses per range:** - 5-15%: 16 responses - 20%: 5 responses - 25-50%: 6 responses - 70-80%: 3 responses |
| **Time allocation**  11. If your answer to the previous question was yes, what tasks were you enabled to allocate your gained time to? (e.g., more exceptional cases, not working overtime etc.) |
| **Tasks where less time was allocated after DP:**   - Measuring tumor depths, not having to take a photo or go to another microscope with a micrometer - Increased measurement accuracy for reporting parameters in less time. - The molecular sessions and the integrated diagnosis of cases are much more agile. - Faster access to cases - Less time spent on large pieces - Generating files for teaching with ease - Working remotely on research topics is easier - Optimizing macroscopy time - Agility to diagnose small samples - No need to look for cases on the trays on my table, enables the pathologist’s work to be more organized - Do not work more hours than necessary     **Tasks where additional time allocation was enabled with DP:**   - "By eliminating valueless tasks, you can allocate more time to difficult cases, investigation, not working more hours than necessary." - Dedicate more time to complex/unusual cases. - Teaching, research - Laboratory management - Being able to look at cases from home (sometimes outside of work hours) - Allows more daily cases to be assessed. - Easier review of old cases, greater accessibility - The flexibility and convenience of having all the patient's images available online improves diagnosis. |
| **Accuracy**  14. If your answer to the previous question was yes, in what way have you achieved increased diagnostic accuracy? (e.g., fewer misdiagnoses, more confidence in the results etc.) |
| **Case Examination Process by the Pathologist**   - Better overview of the slide: allows for the analyzing of the entire sample faster while ensuring that no parts of the slide are left without evaluation - Images are clearer - The possibility of seeing different stains simultaneously allows us to be more certain that each one of them is stained and avoids interpretation errors. - Comparison of slides with controls - Possibility of comparing several slides (notably H&E and IHC on the same screen - Made it easier to ask for second opinions remotely from specialized colleagues - Improved image quality - Measurement and precision - More precision in the measurement of diagnostic parameters: measurement of tumors, counting the number of mitoses, the evaluation of biomarkers (it enables delimiting areas where the pathologists want to count and take notes on the image). - Gives the pathologist more confidence in the results     **Limitations**   - Analysis at x40 magnification is difficult in DP |
| **Patient Outcomes**   - 15. Are you aware if there have been notable improvements in patient care outcomes or treatment decisions attributed to the use of digital pathology? (e.g. enhanced outcomes due to an earlier diagnosis and treatment initiation) |
| - **No:** 16 - **I don't know:** 5 - **Yes:**   - Faster diagnosis in urgent cases   - Use of digital pathology in MDT meetings and sharing images with more specialized clinicians   - In the cases of digital consultations, the second opinion has been accelerated and patients with less frequent/rare types of tumors can more easily have access to highly specialized pathologists   - Earlier treatment for a faster diagnosis and correct staging.   - It allows you to work remotely, if necessary for the patient, outside of working hours, which leads to a decrease in the diagnostic waiting time.   - Faster response to clinicians when more clarification about a specific issue is needed (e.g. easier access to a slide where a clinician wanted more precision on a specific margin)   - Better quantification of bone marrow infiltration in multiple myeloma using computer-assisted counting |
| **Additional Aspects Impacted by DP:**  17. Are there any additional aspects where you saw a positive or negative impact of DP? |
| **Positive:**   - **Collaboration and teaching:** - Being able to share the images in the functional units/multidisciplinary sessions of the hospital and with other centers is very positive. - Positive for teaching, greater comfort in reviewing cases and giving training sessions. - In teaching (for presentations, theoretical classes, etc.). The ability to generate teaching files is much greater with DP. - In resident training. It allows them to write down their doubts and suggestions in the images much more easily, making the teaching process much simpler. - It is perfect for obtaining images of interesting cases, both for students and for presenting them at conferences, publications, etc. There has been a before and after in the time invested in obtaining images and their quality. - **Case examination:** - It is very positive to have immediate access to archived cases (as long as they are not very old). - Greater ease of interpretation of fluorescence in situ hybridization (FISH)-type techniques - Better access to cases, to file cases, and therefore greater comfort at work - Facilitates teleworking. - Makes the specialty more attractive in general     **Negative:**   - Impossibility of performing some diagnostic maneuvers (polarization, direct immunofluorescence...) and less access to previous material (or not digitized, or located on an alternative server) - Specific failures in image scanning interrupt the diagnostic process - On the negative side, if there is a failure in the computer system (electricity, internet, system updates...) the laboratories’ activity is discontinued. - Relies on the hospital network and connection to servers - The dependence on computer failures or errors in the servers slows down the process. - The University does not accept DP as a training tool |
| **Examples of Additional Aspects Impacted by DP:**  19. If your answer to the previous question was yes, can you provide additional examples? |
| - **Positive aspects**      - - **Case Examination and ergonomics:**   - Having the system integrated with the laboratory information system (LIS): access to macro photos, all patient previews and the entire clinical history, digitized archive, and improved traceability.   - Allows immediate comparison with other biopsies from the same patient (the patient's name is displayed next to the WSI on the screen)   - Patient safety and reduction of the risk of error, image and patient reconciliation   - Possibility of displaying several slides in parallel and comparing them, easier to read IHC.   - Easier/faster transition between slides/cases   - The ability to examine at a lower magnification than the microscope   - Wider field of vision   - Changing magnification is faster, without having to focus every time you change magnification   - Easy adaptation   - Saves time from unnecessary tasks (searching for lost slides, organizing them, searching for cases on colleagues' tables)   - Reliable measurements (margin estimation, percentage).   - Image analysis algorithms   - Greater ergonomics during work. Less visual fatigue than when looking through a microscope.   - More comfortable: less eye strain and visual fatigue, seeing digital annotations, increased speed of slide examination   - Possibility to dictate the reports at the same time while looking at the slides on screen      - - **Training, collaboration and flexibility**   - Facilitates the experience of training staff   - Ease of looking at cases together and sharing the case with colleagues   - Ease of taking good quality pictures   - Remote working,   - Ease of slide review meetings in the form of videoconferences, which avoids costly and time-consuming travel   - Research      - **Negative Aspects:**   - When there is an issue with the scanners and the workflow is interrupted   - Images are not very clear in certain cases compared sith the microscope such as counting mitoses but undoubtedly linked to our old generation scanners   - DP with the first generation of scanners are not suitable for certain specialties due to insufficient scanning quality: hematopathology, nephropathology and partly dermato-inflammatory (the microscope is better for the analysis of details)   - Management of both workflows (microscopy and DP) is time-consuming and tiring   - Increased workload for technicians during the transition   - High costs   - No specific coverage   - Increasing workload for the IT team   - “In some specific types of cases (complex surgeries) digital pathology improves the visualization of the lesion and speeds up the diagnostic process. In other types of pieces that require detail, this is lost with digitization.” |
| **Time needed to experience the full impact of DP:**  23. How long after DP implementation did you fully experience its impact on the laboratory’s activity? |
| - **Number of responses per range:** - **Less than 1 month:** 9 responses - **1 month:** 5 responses - **2-3 months:** 5 responses - **6 months to less than 1 year:** 3 responses - **1 to 2 years:** 5 responses - **Ongoing Transition, can't tell:** 5 responses - **I don't know:** 5 responses |
| **Main Barriers for CP implementation on the laboratory level:**  24. What are the most important barriers that pathology departments face currently when it comes to adopting CP/ AI-based pathology solutions? (laboratory level) |
| - **Cost and infrastructure**   - Costs for the use of the algorithm   - Purchasing the algorithm is very expensive and developing them requires a lot of pathologist time to select the areas.   - Infrastructure: computing for connection and storage, IT involvement, lack of bioinformaticians to assist in routine practice   - Implementation and adaptation to the workflow   - Investment in hardware; acquiring and choosing the appropriate equipment, ensuring that the scanning is of adequate quality   - Poorly scanned slides: you need an impeccable technique, which we do not always have. Detection problem on some tissues, such as adipose biopsies. High magnification is generally less efficient, which can impact hematopathology, particularly the analysis of osteomedullary biopsies.   - The consequences of change in other aspects such as pre-analytics      - **Related to the AI tools**    - Validation: poor quality of CP/AI-based evaluations   - AI algorithms are not yet sufficiently trained, they do not correctly detect biomarkers.   - It is a learning path based on trial and error. The algorithms used have not been entirely useful. I would be highly interested, especially in the integration of solutions for 3D reconstructions. - The complexity of pathology makes it difficult to have CP-based tools. Software design should include a collaboration between computer scientists, engineers and pathologists, but this is not feasible in all centers.      - **Human Resources consideration** - Staff training - Willingness to adopt new work methodologies and fear of change - Pathologist reluctance and reconfiguration of processes in the laboratory      - **Other** - As part of a network, the main barrier and at the same time the primarydriver is that these solutions must be conveyed through the network. We cannot independently suggest/purchase one of these solutions. But in an integrated way with the other centers, it could be evaluated and incorporated into the system. |
| **Perceived Benefits of CP**  27. If your laboratory has already implemented a CP tool, what do you think are the benefits of adopting CP? |
| - We have incorporated Ki67 counting for breast cancer. This tool homogenizes the count and makes it more agile. - Currently, only for quantification of biomarkers - Helps in the making of an objective and reproducible diagnosis among colleagues, faster - Precision, reproducibility, time-saving - Objectivity and reproducibility. - Time-saving and improved accuracy - Reproducibility of results, unattractive tasks for the pathologist, reliability and precision of results - More precise evaluations, lower inter- and intra-observer variations - The support of a standardized system - More objective answers, when used correctly and with good AI |
| **Primary Barriers for Wider CP adoption**  28. What are the most important barriers to wider CP adoption? |
| - There is a need for strong scientific evidence and validation of the algorithm. - The difficulty of training algorithms against rare and heterogeneous cases - Connectivity, the level of hardware used and the internet speed - In functional pathology I do not think CP has much use - Primarily economical - Interoperability, resistance to change - There are limited standards - Very few available, well-tested AI tools |

**Table 7 – Number of responders by country (Technicians)**

|  | **UK** | **France** | **Spain** | **Italy** | **Germany** | **Total** |
| --- | --- | --- | --- | --- | --- | --- |
| **Number of laboratories** | 2 | 1 | 3 | 1 | 1 | 8 |
| **Number of Responses** | 10 | 3 | 31 | 1 | 2 | 47 |

**Table 8 – Technicians’ responses to semi-qualitative questions (Likert scale)**

| **Question** | | **DP had a negative impact** | **No, DP did not make any difference** | **DP made a slight/limited positive impact** | **Yes, DP had an important positive impact** |
| --- | --- | --- | --- | --- | --- |
| **Overall workflow** | 4. Did you perceive an improvement in the pathology workflow after the integration of DP? | 1 | 3 | 14 | 29 |
| **Efficiency** | 5. Did you perceive an overall increase in your efficiency after the integration of DP? | 1 | 13 | 10 | 23 |
| **Time allocation to different tasks** | 7. Did DP allow you to allocate your time differently in your current practice/ different tasks? (i.e., do you feel that DP makes you spend more or less time on certain tasks?) | 2 | 12 | 15 | 18 |
|  | **Questions** | **Not difficult** | **Somewhat difficult** | **Difficult** | **Very difficult** |
| **Level of difficulty of the DP learning curve** | 10. How would you describe the learning curve and the steps toward DP integration? (e.g. ease of use of DP equipment, trainings, adaptation to the new workflow…) | 28 | 15 | 3 | 1 |

**Table 9 – Technicians’ responses to qualitative questions (Free text)**

| **Questions and Answers** |
| --- |
| **Efficiency:**  6. If your answer to the previous question was yes, could you provide an estimation in percentage? (e.g., 5-10%, 10-15%) |
| - **Number of responses per range:** - **<10%:** 2 responses - **10 to 25%:** 11 responses - **30-50%:** 8 responses - **60-90%:** 7 responses |
| **Workload:**  8. Did DP increase your workload? |
| **Yes:**   - More time needed for drying slides and scanning (especially if the scanners stop, you need to spend time fixing it) - Need to improve quality of microtome cuts and precision in placing the cut on the slide, and coverslips correctly placed, coverslips or moved labels must be cleaned to prevent scanner failure which takes time - Need to QC slides, cassettes and staining - Increased as it added the step of performing QC on the slides - Time needed for scanner maintenance and calibration, continuous emails with the scanner vendor due to problems with it and our software, new protocols derived from errors in the work chain - Slow recovery of archived cases on the LIS   **No:**   - It has not increased the load, it has removed previous tasks and added new ones such as aligning coverslips, cleaning the slide stainer trays and it has taken away our time from sorting slides by number, case study and pathologist to deliver by hand to the different lockers. What was eaten for what was served. - The workload has been distributed differently |
| **Overall Impact**  9. Are there any additional aspects where you saw a positive or negative impact of DP? |
| - **Positive** - We know when a slide has arrived in the archive because it has been digitized,but even there are cases that are not digitalized correctly, and pathologists directly question technicians asking for explanations and solutions. - The fact of being able to automate many processes due to DP implementation has been very positive. - Yes, despite the inconveniences, the time invested is much less than without DP. you can invest the time in other tasks that were almost impossible to devote to before. - Better workflow, improvements in diagnosis. - You can view any biopsy at any time of the day. - The most positive impact is the reduction in waiting times between cutting a slide, diagnosis by the pathologist, and much faster and more efficient communication between pathologist and technician. - Yes, positive. I have been able to dedicate more time to the quality of service and increase the number of units to serve. - It has made training far easier as it simplifies mixing media types - Yes, for teaching purposes - Easier to QC, also great for training - More time to release cases via the computer in one station (instead of microscope to computer), organizing workflow based on requests and seeing what has been completed via digital capacities. Can release digital urgent cases instead of forcing other sites to wait for slide delivery. - Quality of the sections      - **Negative**: - Slides with too little sample or with agglutinations scan poorly - The negative is that the sheets go directly to the LIS and you cannot see how the cut turned out for each technician. - Machinery and system failures - When equipment fails, work is greatly delayed. - Choice of important consumables because they can have a negative impact on the DP - Although efficiencies were seen for pathologists, it did increase the time taken to QC work for the laboratory - There is no need for manual application time, but the dyes cannot be checked for functionality in a timely manner. This may lead to delays. - Scanning time is too long - "There are positive and negative aspects. For all these reasons, the technicians' time spent on the process of cutting the biopsy has not been reduced, but we now have to dedicate it to aspects that we did not spend time on and with greater attention. Quality control is much more meticulous. You have to dedicate more time to remaking a block, cutting it, fishing it, dedication to processors, cutters, dyers…" |
| **Time needed to experience the full impact of DP:**  11. How long after DP implementation did you fully experience its impact on the laboratory’s activity? |
| - **Immediately:** 9 responses   - **<3 months:** 1 response   - **>3 months and <1 year**: 15 responses   - **1 year:** 6 responses   - **>1 year:** 2 responses   - **DP transition ongoing:** 2 responses   - **I don't know:** 4 responses      - "Almost immediately because you have to think of things differently. Instead of a person you have to work in a way that works with the algorithm the scanner uses to gain the best success rate."   - “The impact is immediate, since it changes the workflow in many aspects.”   - “It is easy to learn if you want to learn, but many people are not willing to accept the changes that must be made in their way of working to promote good and rapid digitalization.”   - A few months. It was a workflow issue at first and getting the settings right for scanning to ensure tissue is in focus. But once the maintenance, cleaning, and QC workflow were adjusted, it became consistently smooth   - We continue in learning mode, since we are a diverse team, with a wide number of functions and it has been implemented during the holiday period. |

## Appendix 4 – Detailed overview of the NPV model

**Table 10 – Summary of the NPV model features and assumptions (Free text)**

| Category | Description |
| --- | --- |
| NPV Calculation | The model evaluates the financial and operational implications of DP adoption by focusing on the Net Present Value (NPV) over a 7-year forecast period, assessing long-term financial gains such as increased revenue and efficiency to justify costs. Factors unrelated to DP transition are excluded. |
| Scope Of Analysis | Includes 7 leading pathology departments, with 81.4% (range: 44.5%-100%) of cases digitized. It considers case volumes, slides processed, turnaround times, reimbursements, personnel metrics, infrastructure investments, and scanner requirements. |
| Assessed Changes | By isolating DP-related changes, it assesses whether long-term financial benefits justify significant initial and recurring costs. The model provides a transparent framework for evaluating DP's financial feasibility, highlighting critical variables and scenarios influencing success. |
| Forecasted Period | 7-year period, following the best practices outlined by [Koller T. et al., 2020,](https://www.wiley.com/en-it/Valuation%3A+Measuring+and+Managing+the+Value+of+Companies%2C+7th+Edition-p-9781119610885) who recommend this timeframe to capture the long-term financial implications of substantial investments. |
| Data Source | Includes pre-DP implementation data collected during on-site visit data as well as during offline alignments. Parameters like productivity gains, reimbursement tariffs, and scanner utilization rates are based on primary interviews and field observations. |
| Investigated Cases | The best- and worst-case scenarios were determined using the highest and lowest 20 percentiles of the data distribution, respectively. In cases where sufficient data were unavailable to calculate percentiles, the average was used as an alternative measure. |
| Assumptions | This model focuses exclusively on variations directly attributable to Digital Pathology (DP) implementation, relying on baseline data collected before its adoption. By isolating these changes, the model provides an assessment of specific investments and benefits related to the transition. Where feasible, data on benefits and required investments spanning at least three years prior to DP implementation were collected, extending to the time of on-site visits. Current-year data were extrapolated based on observed trends, assuming linear growth. |
| Scanner Requirements | Scanner requirements were adjusted based on case volume changes, using 75,000 slides processed annually per scanner as a baseline, consistently with what has been observed during laboratory visits. This figure reflects the diverse scanner capacities across laboratories and includes an average of 30% free capacity to accommodate anticipated demand growth. |
| Economic Metrics | The model computes the increase in slide volume by applying productivity gains directly to slides analyzed rather than cases. This approach accounts for DP's primary goal of streamlining slide handling, which inherently results in higher case analysis volumes. The number of cases was converted into corresponding slide numbers using a coefficient derived during on-site visits, which determined an average of six slides per case. Additionally, translating the benefit of increased slide numbers using case-based reimbursement tariffs provides consistent and practical financial estimations. No dedicated reimbursement code exists for DP yet in the countries included in the analysis. The potential benefit arising from an increase in volume therefore takes only into account existing tariffs collected through primary interviews. Potential future changes in the reimbursement structure through dedicated coding might result in increased upside benefit for DP, which are not included in our current model as they are unforeseeable at the time of writing. |
| Primary And Secondary Consultations Productivity Increase | Increases in productivity and case volumes, encompassing primary cases and secondary consultations, were calculated as the percentage variation in cases processed before and after DP implementation. Increase in productivity and case volume was calculated as the percentage variation in cases processed before and after DP implementation. The average number of cases processed - and secondary consultation - before DP implementation was used as the baseline to calculate the increase, and the average number of cases – and secondary consultations - processed after DP was calculated to analyze the variation. The baseline for this calculation was the average number of cases processed pre-DP, with post-DP averages analyzed to quantify variations. The observed growth post-DP implementation was 9.46%, while natural growth, estimated at 2.06%, was extrapolated from this pre-DP parameter. Therefore, the calculated productivity improvement was determined by the difference between 9.46% and 2.06%, resulting in 7.40. The natural growth aligns with trends reported on the WHO Website ([WHO Website, Visited 11/19/2024](https://www.who.int/news/item/01-02-2024-global-cancer-burden-growing--amidst-mounting-need-for-services)). The productivity increase has only been adjusted for the impact of natural growth, as secondary consultations may primarily benefit from the increase due to improved, primarily informal, communication among pathologists. |
| Limitations | - To ensure precision, the model excludes unrelated tariffs, such as those tied to inpatient settings, and outliers that could distort findings.  - Depreciation and amortization, as they are irrelevant to business case evaluations.  - Commercial partnership benefits (e.g., AI collaborations) except in best-case scenarios due to limited applicability. |
| Commercial Partnerships | Observed in one context and included only in best-case scenarios. Benefits may arise from collaborations with AI developers or pharmaceutical companies but involve complexities such as data privacy compliance. |
| Discount Rate | A 5% discount factor was applied throughout the analysis, aligning with standard financial modeling practices to account for the time value of money ([Lars Hultkrantz, 2021)](https://link.springer.com/article/10.1007/s10198-020-01257-x?utm_source)). |
| Interactive Model Features | The model has been designed as a tool for pathologists to evaluate the feasibility of DP investments and their long-term benefits. Users can adjust parameters such as: Percentage of cases digitized; Case processed, slides per case, productivity increases; revenues from big data and partnerships; visualization software costs; scanner capacities and prices; staffing and infrastructure costs. The default values suggested by the authors provide a starting point, while user-friendly features allow for customization without requiring advanced financial or economic expertise. |

**Table 11 – List of key inputs (Base, Best and Worst Cases)**

| Parameter | Base Case | Best Case | Worst Case |
| --- | --- | --- | --- |
| Case Volume Growth | Annual growth of 2.06% ([WHO Website, Visited 11/19/2024](https://www.who.int/news/item/01-02-2024-global-cancer-burden-growing--amidst-mounting-need-for-services)), baseline 56,061 cases | Same growth rate applied | Same growth rate applied |
| Slides Processed | Baseline of 273,156 slides | Same baseline applied | Same baseline applied |
| Productivity Increase | 7.40% | 14.43% | 2.59% |
| Reimbursement Tariffs | €58 (public), €60.58 (private) | €64 (public), €73.40 (private) | €53 (public), €53 (private) |
| Public Case Share | 97.80% | 100.00% | 93.84% |
| FTE Redistribution - Technicians | 0.80 annually | 1.00 annually | 0.00 annually |
| FTE Redistribution – Pathologists | 0.20 annually | 0.60 annually | 0.00 annually |
| Salary – Pathologists  (1 FTE) | €78,350 annually | €78,350 annually | €78,350 annually |
| Salary – Technicians  (1 FTE) | €49,217 annually | €49,217 annually | €49,217 annually |
| Initial Scanner Investment | €263,479 each, 5 scanners | 240,000 each, 5 scanners | 382,134 each, 5 scanners |
| Scanner Maintenance | €65,085 annually | €65,085 annually | €65,085 annually |
| It Infrastructure Costs | Initial: €278,034, Annual: €155,000 | Initial: €240,000, Annual: €155,000 | Initial: €382,134, Annual: €155,000 |
| Workstation Cost | €4,211 each | €830 each | €9,588 |
| Software Costs (Annual) | Pathology Viewer: €73,613; LIS: €136,895 | Pathology Viewer: €62,571; LIS: €136,895 | Pathology Viewer: €84,655; LIS: €136,895 |
| Software Initial Setup Costs | Pathology Viewer: €103,875;  LIS: €172,419 | Pathology Viewer: €88,294; LIS: €72,842 | Pathology Viewer: €119,456; LIS: €339,754 |
| Microscope Replacement Savings | €796 per unit annually, 6 units reduced | €796 per unit annually, 6 units reduced | €796 per unit annually, 6 units reduced |
| Secondary Consultation Volumes | 2,785 | 10,557 | 2,785 |
| Big Data and Commercial Partnerships Revenue | Excluded in base case | €900,000 annually included | Excluded in worst case |
| NPV (7 Year) | €204,927 | €9,905,149 | (€3,237,199) |

**Table 12- List of outputs trends (Base, Best and Worst Cases)**

| Parameter | Base Case | Best Case | Worst Case |
| --- | --- | --- | --- |
| Initial Case Volumes | 56,061 cases | 56,061 cases | 56,061 cases |
| Case Volume Growth | 7.40% annually, reaching 75,201 cases by Year 7 | 14.4% annually, reaching 117,213 cases by Year 7 | 2.1% annually, reaching 64,662 cases by Year 7 |
| Whole-Slide Images (WSIs) Processed | 273,156 at baseline, increasing to 450,210 slides by Year 7 | 701,729 slides by Year 7 | 326,723 slides by Year 7 |
| Scanner Deployment | 5 scanners initially; 1 scanner added in Years 5 and 7, for a total of 7 scanners by Year 7 | 5 scanners initially; 1 added in Year 1, 1 in Years 3, 5, 6 and 7 for a total of 10 scanners by Year 7 | 5 scanners initially and 0 added through Year 7 |

***Fig.4.*** *Discounted Cash Flow (Base, Worst, and Best Scenario) - Applied Discount Rate: 5.00%*

***Fig. 5.*** *Cumulative Discounted Cash Flow (Base, Worst, and Best Scenario) - Applied Discount Rate: 5.00%*

***Fig.6.*** *Sensitivity Analysis – Variation vs. Base Case NPV*
